# Classification Metrics for Improved Atmospheric Correction of Multispectral VNIR Imagery

**DOI:** 10.3390/s8116999

**Published:** 2008-11-05

**Authors:** Rudolf Richter

**Affiliations:** German Aerospace Center (DLR), Remote Sensing Data Center (DFD), Münchener Str. 20, D-82234 Wessling, Germany; E-mail: rudolf.richter@dlr.de

**Keywords:** Aerosol retrieval, surface reflectance, haze, cloud, water

## Abstract

Multispectral visible/near-infrared (VNIR) earth observation satellites, e.g., Ikonos, Quickbird, ALOS AVNIR-2, and DMC, usually acquire imagery in a few (3 – 5) spectral bands. Atmospheric correction is a challenging task for these images because the standard methods require at least one shortwave infrared band (around 1.6 or 2.2 μm) or hyperspectral instruments to derive the aerosol optical thickness. New classification metrics for defining cloud, cloud over water, haze, water, and saturation are presented to achieve improvements for an automatic processing system. The background is an ESA contract for the development of a prototype atmospheric processor for the optical payload AVNIR-2 on the ALOS platform.

## 1. Introduction

Although satellite images from Earth-observing multispectral VNIR instruments cover only a part (450 – 900 nm) of the solar reflective spectrum (400 – 2500 nm) they contain a rich source of spatial and spectral information about the Earth's surface. Typical application fields are urban developments, agriculture, forestry, bathymetry, coastal zone mapping, monitoring, and change detection [1-5].

**Figure 1.**
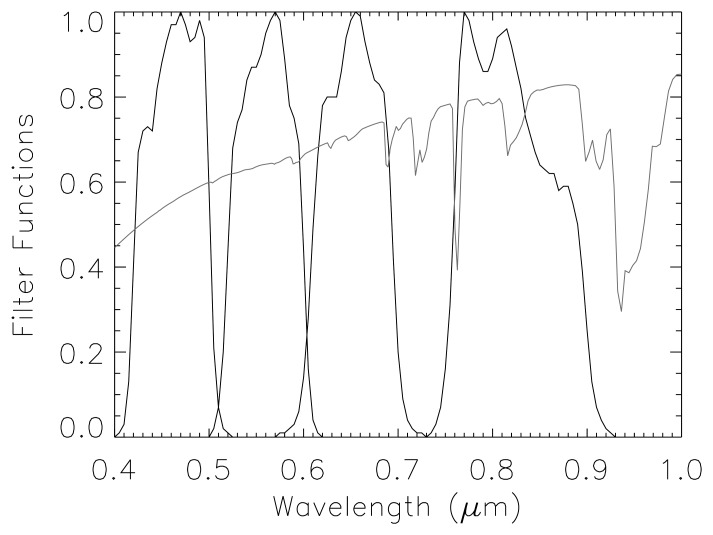
AVNIR-2 channel filter curves and atmospheric transmittance.

A disadvantage of these systems is the small number of spectral bands, but the plus side contains strong arguments such as lower costs and a higher spatial resolution. So these instruments will not become obsolete with the advent of future hyperspectral sensors, but will rather complement each other, e.g. during evaluation and validation of coarser spatial resolution measurements. Therefore, it is worthwhile to improve atmospheric correction (AC) algorithms for multispectral VNIR imagery. An essential part of AC methods is a classification of the scene into cloud, cloud over water, haze, and saturated pixels. This contribution presents new and improved metrics for defining these masks in the context of an existing AC algorithm. Selected results are shown employing ALOS AVNIR-2 data [6]. It is a sensor with four VNIR channels (see Figure 1) and a nadir spatial resolution of 10 m. However, the proposed method is generally applicable to this type of instrument and not restricted to AVNIR-2. The ALOS platform was successfully launched by JAXA (Japanese Aerospace Exploration Agency) on January 24 2006, and also contains the panchromatic PRISM camera for stereo mapping and the PALSAR (phased array L-band synthetic aperture radar).

ALOS data will be provided to European and African users through a cooperative agreement between ESA and JAXA. Since JAXA does not deliver atmospherically corrected AVNIR-2 imagery, ESA decided that such a product should also be available to customers and contracted DLR to develop prototype processors for AVNIR-2 on ESA's ground stations, i.e.; level 1 (radiometrically corrected and orthorectified products), and level 2 (surface reflectance). The latter part is described here.

The radiometric calibration uses a linear equation with offset and gain to transform the recorded image digital numbers into the corresponding at-sensor radiances. These coefficients may change with time and geographical position, and they are included in the image meta file.

## 2. Method

The atmospheric correction method is based on radiative transfer calculations with the MODTRAN4 code [7]. A comprehensive set of calculations is performed for different atmospheric conditions, solar and view geometries. Results are stored in a database of look-up tables (LUTs). These high-spectral resolution LUTs are then resampled with the sensor-specific channel filter curves, and the atmospheric correction can be conducted as described in [8, 9]. The usual Lambertian assumption is employed for the surface reflectance law, and the standard rural (continental) aerosol model [10] is selected, because a reliable estimate of the aerosol type over land is not possible with a few VNIR channels.

The standard AC procedure starts with the derivation of the aerosol optical thickness (AOT) at 550 nm followed by the surface reflectance retrieval [11-14]. The influence of the atmospheric water vapour column (W) is very small for typical VNIR sensors, compare Figure 1, and since the water vapour cannot be derived from this type of instrument, a typical seasonal value has to be taken. The corresponding sensitivity and small errors were calculated with MODTRAN4 and are summarized in Table 1 for a vegetation and soil surface. The error is usually below 1% reflectance, and can be up to 1.8% reflectance in the NIR for high reflectance values of 40% and a large error (factor 2) in the W estimate. So a reasonable seasonal estimate of W should yield smaller errors than given in Table 1. There is a small effect even in band 1, although this band is free of atmospheric water vapour absorption. The reason is that a change of air humidity changes the aerosol scattering properties, i.e. the aerosol path radiance.

The standard aerosol retrieval techniques are based on the detection of dark reference pixels in the scene and either use short-wave IR bands around 1.6 or 2.2 μm and spectral correlations with visible bands [11] or many VNIR bands [12, 13]. Therefore, they cannot be applied to VNIR instruments with only a few spectral bands. An empirical method was published for these cases which worked satisfactorily [14].

### 2.1. New Method

The basic method employs the AC technique described in [14], but includes a number of additional classification rules to partition the scene into cloud, cloud over water, haze, and water. First, the visibility map is iterated, checking the surface reflectance values of water bodies in the scene. If the reflectance of the water pixels is negative, the visibility is raised (aerosol optical thickness AOT decreased) until the reflectance is greater than zero or the maximum number of iterations is reached. If the scene contains no dark reference areas and no water bodies, i.e.; desert regions, it will be processed with a constant visibility of 23 km or a typical climatological value. Since desert areas have high surface reflectance values, typically ranging from 0.1 to 0.5 in the VNIR, retrieval results do not critically depend on the assumed visibility. As an example, AVNIR-2 surface reflectance values from the Libyan desert calculated with VIS=23 km and VIS=50 km differ less than 0.01 reflectance units. Aerosol information from other sources, e.g. AERONET or MODIS products, can also be employed, but the objective is a fast autonomous performance independent of other processing chains. Other improvements are a set of physically based metrics for the masking of cloud, haze, and water as discussed below. These rules are formulated as *surface reflectance* or *top-of-atmosphere (TOA) reflectance* thresholds in the available VNIR bands. It must be emphasized that these thresholds are set based on the pertaining physical reflectance signatures (cloud, water), yet there is always some arbitrariness in thresholds. It is also clear that a set of a few VNIR thresholds cannot uniquely characterize cloud, haze, and water for all situations, for instance, cloud spectra can be similar to sand, so these two classes may mix. On the other hand, deep water and shallow water over sand exhibit very different spectral behaviour, and a certain water mask might capture deep water, but miss shallow water.

Nevertheless, the proposed rules improve the AC results in many cases as demonstrated with AVNIR-2 imagery in Section 3.

The following rules are used to generate masks of cloud, haze, and water. These masks are calculated as a first step before the AC starts. They are important for the quality of AC, because for instance the haze removal over land relies on rather accurate masks of haze, cloud and water. The cloud mask employs a certain TOA reflectance threshold (e.g.; *T_1_* = 0.3) in the blue channel, and a condition that restricts the blue-to-NIR reflectance slope from being overly positive:

(1) $$\begin{array}{ccc} \rho_{\text{blue}}^{*}=\frac{\pi \;L_{\text{blue}}}{E_{\text{blue}}\;\text{cos}(\theta_{s})}>T_{1} & \text{and} & 0.8\;\rho_{\text{blue}}^{*}<\rho_{\text{NIR}}^{*}<1.2\;\rho_{\text{blue}}^{*} \end{array}$$

where the asterisk indicates TOA reflectance, blue is the index for a blue spectral channel, and *L*, *E*, and θ_s_ denote the TOA radiance, extraterrestrial solar irradiance, and solar zenith angle, respectively. If the VNIR instrument has no blue channel, the green channel is used as a substitute. The second part of the relationship (1) states that the TOA reflectance from the blue to the NIR channel must stay approximately constant (deviations below 20%), because clouds usually exhibit a spectrally flat behaviour [15]. As mentioned before, this cloud criterion might still mix with surface types such as bright sand or soil although these tend to have a reflectance increase in the VNIR spectrum [16-18]. The threshold *T_1_* = 0.3 might be raised depending on geography (e.g. in desert regions with bright sand), but ambiguities can still remain. For winter scenes with snow cover, the relationships (1) might also fail, because snow has a high reflectance in the blue-to-red spectrum, decreasing with wavelength [19].

As demonstrated in Section 3, thin and medium thickness clouds over water can often be captured with the two criteria:

(2) $$\begin{array}{ccc} 0.2\leq \rho_{\text{blue}}^{*}<0.4 & \text{and} & \frac{d\rho^{*}(\lambda )}{d\lambda }<0 \end{array}$$

The second part of relationship (2) describes a negative gradient of the TOA reflectance, i.e.; ρ* decreases from the blue to the NIR wavelengths. This property is employed because the reflectance spectrum of clouds is usually flat, but the Rayleigh and aerosol scattering above a dark background will increase the apparent reflectance in the shorter wavelength bands, causing a negative gradient. The haze level map (over land) is calculated in five steps and details can be found in the references [20, 21]. First, the haze / clear mask is computed with the haze component of the tasseled cap (TC) transformation [22]. If μ(TC) denotes the mean of the haze TC map, an empirical global threshold T is defined as T = μ(TC). Then, clear and haze pixels are defined as pixels with TC(clear) ≤ T and TC(haze) > T, respectively. Additionally, clear pixels must not belong to the cloud and water mask. Secondly, the scatterplot of the blue and red channel digital numbers is calculated for the clear pixels and the slope *α* of the linear regression line defines the “clear line” corresponding to the clear part of the scene. The next step is the evaluation of the haze level map H, a transformation orthogonal to the “clear line”

(3) $$H=DN_{1}\sin\alpha -DN_{2}\cos\alpha$$

where DN_1_ and *DN_2_* are the digital numbers in the blue and red band, respectively. Again, if no blue band exists, the green band is taken as a substitute. No attempt is made to introduce a *haze-over-water* mask as this haze cannot be removed satisfactorily. The fourth step is the per channel and per haze level calculation of the histograms of the clear and haze areas, and the last part computes the difference DN(haze) – DN(clear) and subtracts it from the corresponding haze pixels on the original DN level, i.e. before the AC algorithm starts. Note: Equation (3) and the TC transformation are not a metric relationship because they deal with the sensor-specific digital numbers. However, the haze correction is executed on the DN level before any AC algorithm is applied, and therefore it is still adapted to each sensor and valid for any type of VNIR instrument.

In the absence of SWIR channels the water mask is usually defined by a threshold in the NIR band. Clear water has very low surface reflectance values in the 850 nm region, typically *ρ*(NIR) < 0.02. However, turbid water may have higher surface reflectance values, so there is no unique choice for a NIR threshold. As a compromise, a single threshold of *ρ*(NIR) = 0.05 was used in the past to mask water bodies for VNIR sensors [23]. This criterion is not unique as surfaces in the shadow may yield similar apparent reflectance values. A threshold for the surface reflectance in the blue or green channel is even more difficult, because of the multitude of possible water constituents, concentrations and the influence of the shallow water body bottom reflectance. Instead of the single threshold for the *surface reflectance ρ*(NIR) = 0.05, a new approach was successfully tested using the *TOA reflectance gradient*:

(4) $$\begin{array}{ccc} \rho_{\text{blue}}^{*}<0.2 & \text{and} & \frac{d\rho^{*}(\lambda )}{d\lambda }<0 \end{array}$$

The first part of the relationship (4) will include shallow water over bright sands in the water mask, compare section 3. The second part mimics the typical behaviour of a negative *TOA reflectance* gradient in the blue to NIR region. It is essential here to employ the TOA reflectance ρ* and not the *surface reflectance ρ* because *ρ(blue*, *water)* < *ρ(green, water)* as well as *ρ(blue*, *water)* > *ρ(green, water)* might happen, depending on water constituents. However, because the path radiance is greater in the blue than in the green band, we obtain ρ*(blue,water) > ρ*(green,water) in the majority of cases. Section 3 will demonstrate some examples of the superior performance of the relations (4) compared to the single threshold of *ρ*(NIR) = 0.05.

Saturated pixels have to be identified separately, because the retrieved reflectance spectrum will contain artifacts. For sensors with an 8 bit/pixel radiometric encoding, saturated pixels are flagged if the digital number is close to or equal to the maximum value (DN=255). The optical design of sensors is adjusted to the maximum expected radiance in each channel which in turn depends on the solar and viewing geometry and surface reflectance. The expected maximum surface reflectance of different surface types (soil, sand, vegetation, water) is smaller in the blue band than in the remaining part of the VNIR spectrum (except for snow). So the critical situation of saturation (caused by a higher surface reflectance than assumed in the design, e.g.; due to bidirectional or specular reflection) usually occurs in the shortest wavelength band (blue or green), and this is also true for snow. Therefore, only this channel is checked for the initial saturation mask to save time. However, during the channel processing loop the percentage of saturated pixels is evaluated for each channel and this documentation is put into a “log” file containing useful information, warnings, and errors.

## 3. Selected Results

Only three processing examples will be shown to keep the scope of the paper within reasonable limits, but they cover the main problems inherent in atmospheric correction of VNIR imagery. The first example treats the haze removal and demonstrates the superior results of calculating the water mask with the relationships (4) instead of the simple reflectance criterion ρ(NIR, water) < 0.05.

The second example investigates problems with saturated pixels, and which spectral band is most sensitive concerning saturation.

The third example treats cloud over land, over water, and coastal regions with very high water reflectance values in the visible spectrum. Although the atmospheric correction is dedicated to land surfaces it is quite useful to obtain realistic masks of water and cloud. One reason is an improved performance of the haze removal (as shown in the first example), the other reason is a more accurate treatment of the adjacency effect.

Since clouds have high reflectance values, but are not located at the ground level, the usual adjacency correction has to be modified. Because of the higher altitude of clouds and the reduced air volume for scattering, they do not contribute as much to the adjacency effect as ground surfaces of the same reflectance. Therefore, as an approximation, cloud reflectance values are replaced with the average non-cloud surface reflectance (per channel) during the adjacency calculation. Of course, afterward the original reflectance is assigned back to cloud pixels.

### 3.1. Scene with haze

**Figure 2.**
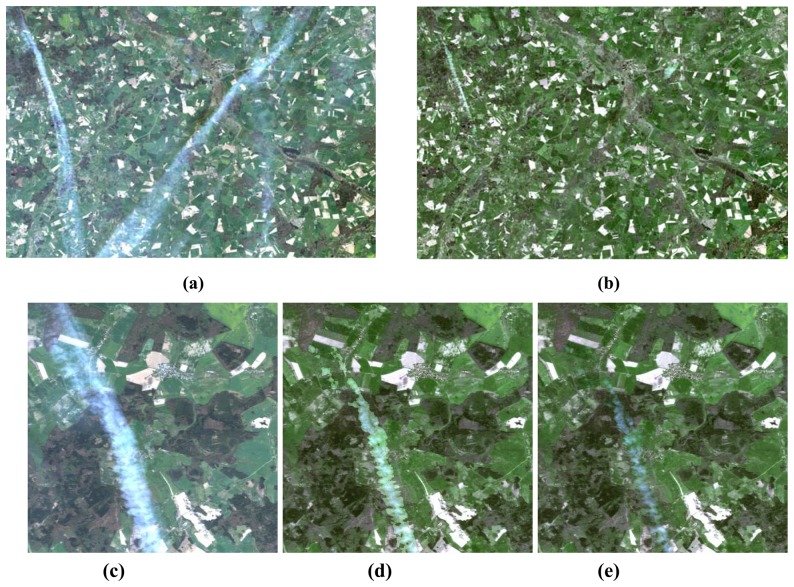
(a) original scene RBG=3,2,1, (b) after atmospheric correction with haze removal; (c) zoom of (a); (d) zoom of (b) with simple water mask, i.e. ρ(NIR)=0.05; (e) same as (d) but with improved water mask, i.e.; relationships in (4).

Figure 2 presents a true color subset (RGB = bands 3, 2, 1) of an AVNIR-2 scene acquired 16 April 2007 over northern Germany with thin and thick haze clearly visible over different areas. The image was recorded with an off-nadir pointing angle of 41°, but not in the principal plane. The top right (b) presents results of the atmospheric correction and additional haze removal employing the simple water mask with the surface reflectance threshold criterion ρ(NIR)=0.05. The whole scene contains large water bodies (not shown in this subscene), so an accurate calculation of the water mask should improve the quality of the haze removal. The bottom part (c) shows a zoomed view from the top left part, again original data. Zoom (d) presents the corresponding area of processing (b), while zoom (e) is based on calculations with the improved water mask of relationship (4) that correctly classifies turbid water escaping the simple criterion ρ(NIR)=0.05. Although the haze removal in (d) is done well, the results of (e) are superior in regions with thick haze.

### 3.2. Scene with saturated pixels

**Figure 3.**
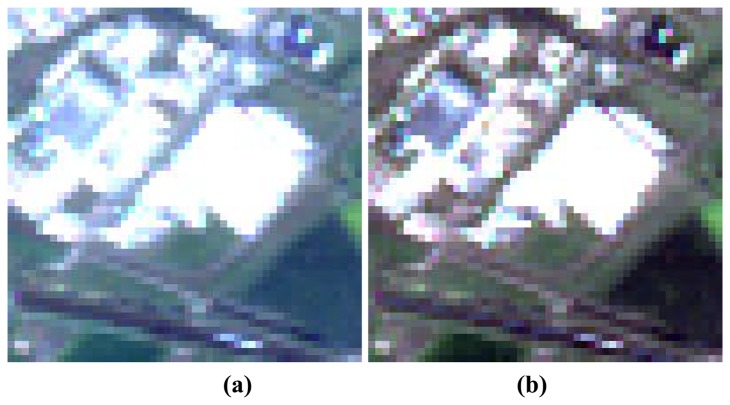

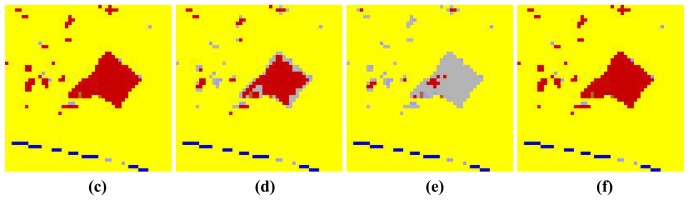
(a) original scene RBG=3,2,1; (b) after atmospheric correction with haze removal. Red coding in (c) to (f) represents the saturated pixels in bands 1, 2, 3, and bands 1+2+3, respectively.

Figure 3 shows a subset of another AVNIR-2 scene recorded over Germany, 16 April 2007. It features buildings and a parking lot of high reflectance. Similar to Figure 2, the scene was taken with an off-nadir pointing angle of 41°, but not in the principal plane. Since it is a hazy scene (Figure 3a), the atmospheric correction with an additional haze removal was applied (Figure 3b). The bottom images represent the map of haze (coded yellow), cloud (grey), water (blue), and saturated (red) pixels. Saturation usually occurs in the visible bands because most surfaces have low-to-medium reflectance (ρ < 0.3) values there (except snow and bright sands), and the instrument's gain setting is adjusted to resolve the corresponding radiance range. The NIR band has to handle high reflectance values (ρ=0.4 – 0.7) because of vegetation canopies, therefore saturation in the NIR is seldom a problem.

### 3.3. Coastal scene: bright sand and cloud, cloud over water

**Figure 4.**
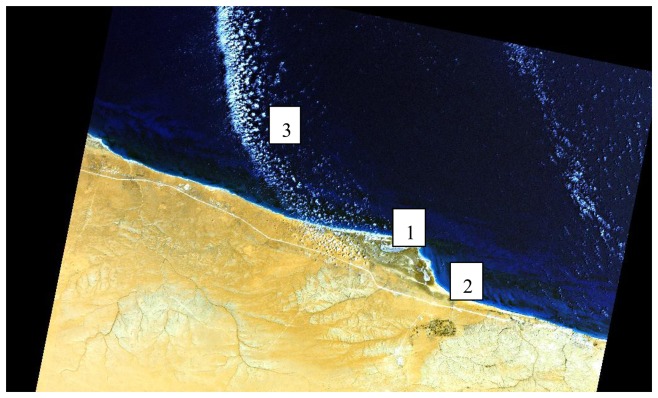
Coastal scene of Libya, RGB=bands 3, 2, 1.

Figure 4 shows part of a coastal scene of Libya, recorded with a nadir view on 24 April 2007. The labels 1, 2, 3 indicate the vicinity of three areas that are discussed in detail with zoomed images.

The haze/cloud/water/saturation masks (c to f) demonstrate that the blue band contains most saturated pixels, followed by the green band, due to the commonly employed gain setting strategy. Therefore, the automatic processor will check only the blue band concerning saturation. This enables a faster processing, and saturation in one band is enough to flag erroneous surface reflectance spectra. Note that the usage of the blue band to determine saturation (instead of the green or red band) also reduces the likelihood of these pixels being classified as cloud.

**Figure 5.**
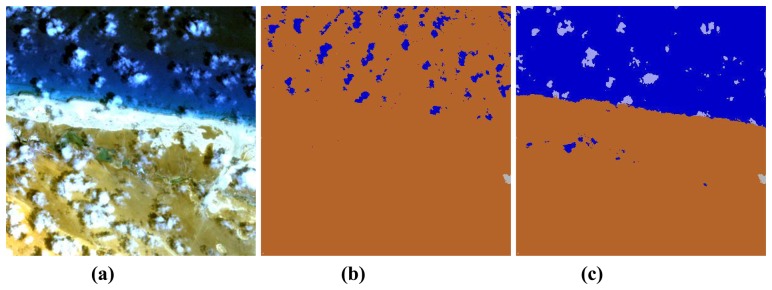
Zoom of Figure 4, around label 1, and classified maps, see text.

Figure 5 presents a zoom area below label 1. The beach area consists of bright sand and a coastal zone of flat water over sand appearing in bright blue in the true color image. Figure 5 (b) represents the cloud (grey) / water (blue) / land (brown) mask where the water pixels are determined with the surface reflectance criterion ρ(NIR) < 0.05 and no criterion for cloud over water. The cloud threshold is set at ρ*(blue)=0.3. Obviously, all clouds over water have ρ*(blue) < 0.3 in this scene, and therefore no clouds over water can be found.

**Figure 6.**
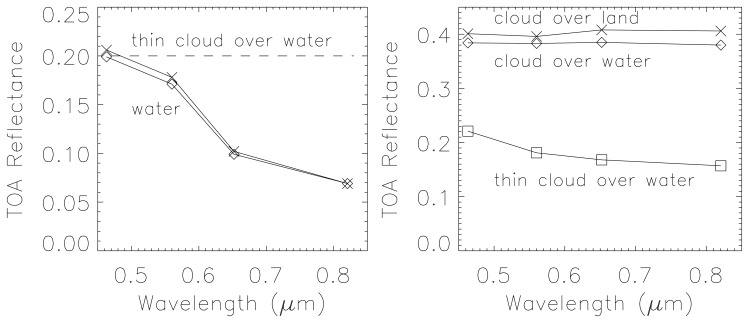
TOA reflectance spectra of cloud, cloud over water, and water from Figure 5.

Additionally, only a few water pixels are correctly assigned in Figure 5(b). Figure 5(c) achieves a better classification of land, water, and cloud over water (coded grey-blue) by using the relationships (2) and (4). However, the scattered clouds over land are not recognized in both cases (Figures 5b,c, except for a few pixels at the lower right image border) because their signature is quite similar to the bright sand. The addition of a spatial context algorithm might improve the classification which is a future option if an overall near real time performance can be achieved. Also, the spectral differentiation between thin clouds over water and bright water is very difficult for this scene, see the corresponding spectra in Figure 6. As an example, Figure 6 (left) shows a TOA spectrum of water (diamond symbol) and thin cloud over water (× symbol). These were visually verified by inspecting the neighborhood at two spots. However, the difference in their TOA spectrum is smaller than 0.01, so if the water underground were slightly higher than the threshold ρ*(blue) = 0.2 the classification would erroneously assign the ‘cloud over water’ class instead of ‘water’.

**Figure 7.**
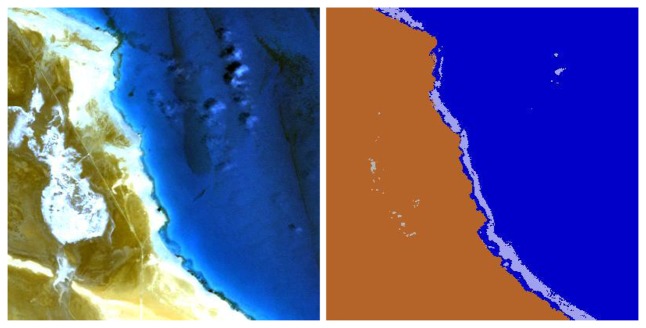
Bright coastal water with spectrum similar to ‘cloud over water’.

Similarly, Figure 6 (right) demonstrates the difficulty of correctly assigning the ‘cloud over land’ class and the ‘cloud over water’. Obviously, an unambiguous class assignment with a few spectral channels is not possible as demonstrated with Figure 7 from the area close to label 2 of Figure 4. Here, the bright coastal water very close to the land/water boundary is misclassified as ‘cloud over water’ (coded grey-blue).

**Figure 8.**
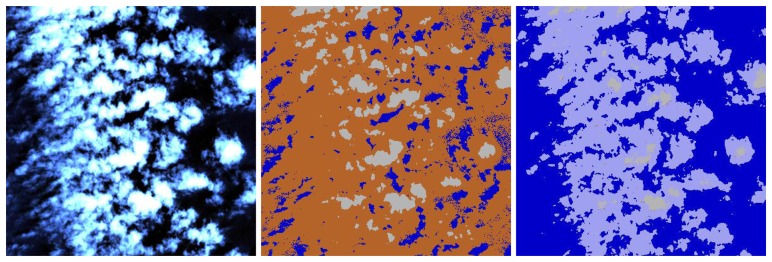
Water and cloud over water (area with label 3 of Figure 4).

Nevertheless, these problems have to be expected, and Figure 8 (area near label 3 of Figure 4) shows that the overall assessment of cloud (over land), cloud over water, and water can be considered as satisfactory for VNIR sensor imagery.

The left part of Figure 8 shows the original scene (RBG=bands 3,2,1), the central part a classification with the cloud threshold ρ*(blue) > 0.3 and water: ρ(NIR) < 0.05 where most cloud over water pixels are assigned to the land class. The right part presents the map obtained with the relationships (2) and (4) for the classes ‘cloud over water’ and ‘water’, respectively. A substantial improvement is obtained with the newly defined set of rules.

## 4. Summary and Conclusions

A new classification metrics has been presented to improve atmospheric correction over land for multispectral VNIR instruments with only a few channels (typically 3 – 5). The method can be used for the automatic processing of imagery without additional information from other satellites or weather stations. A “by-product” of the method is a map of cloud/haze/water and saturated pixels. It is a fast method (90 sec execution time for a 7,000 × 7,000 pixel scene with 4 channels, Intel 2.66 GHz processor, Linux operating system) suitable for the processing in satellite ground stations and it works exclusively with spectral criteria on a per-pixel basis. The proposed technique fails if spectral ambiguities exist. In this case, the largest errors in the surface reflectance retrieval are likely for the haze affected areas, because the quality of the haze removal depends critically on the derived cloud and water masks. Nevertheless, successful tests have been demonstrated for AVNIR-2 data in the majority of cases with this prototype processor.

## Figures and Tables

**Table 1.** Surface reflectance retrieval error Δρ (percent reflectance) for W=0.8 and 4.1 cm. The true water vapour column is W=2.0 cm.

**AVNIR-2 band**	**Vegetation:ρ** = **(2.7, 4.8, 2.8, 41.4)%**		**Soil:ρ** = **(5.2, 9.5, 10.4, 23.3)%**	

	Δ**ρ (W**=**0.8 cm)**	Δ**ρ (W**=**4.1 cm)**	Δ**ρ (W**=**0.8 cm)**	Δ**ρ (W**=**4.1 cm)**

1	-0.3	+0.1	-0.3	-0.5
2	-0.3	+0.1	-0.2	-0.1
3	-0.3	0	0	-0.1
4	+1.7	-1.8	+0.8	-1.3
